# Molecular Mechanisms and Function Prediction of Long Noncoding RNA

**DOI:** 10.1100/2012/541786

**Published:** 2012-12-23

**Authors:** Handong Ma, Yun Hao, Xinran Dong, Qingtian Gong, Jingqi Chen, Jifeng Zhang, Weidong Tian

**Affiliations:** Institute of Biostatistics, School of Life Science, Fudan University, 220 Handan Road, Shanghai 2004333, China

## Abstract

The central dogma of gene expression considers RNA as the carrier of genetic information from DNA to protein. However, it has become more and more clear that RNA plays more important roles than simply being the information carrier. Recently, whole genome transcriptomic analyses have identified large numbers of dynamically expressed long noncoding RNAs (lncRNAs), many of which are involved in a variety of biological functions. Even so, the functions and molecular mechanisms of most lncRNAs still remain elusive. Therefore, it is necessary to develop computational methods to predict the function of lncRNAs in order to accelerate the study of lncRNAs. Here, we review the recent progress in the identification of lncRNAs, the molecular functions and mechanisms of lncRNAs, and the computational methods for predicting the function of lncRNAs.

## 1. Introduction

Proteins and related protein-coding genes have been the main subject of biological studies for years. However, with the development of RNA sequencing technology and computational methods for assembling the transcriptome, it has become clear that besides protein-coding genes much of the mammalian genome is transcribed, and many noncoding RNA (ncRNA) transcripts tend to play important roles in a variety of biological processes. Understanding the function of ncRNAs has become one of the most important goals of modern biological studies [1–3]. ncRNAs can be classified into several distinct subclasses, including processed small RNAs [4], promoter-associated RNAs [5], and functional long noncoding RNAs (lncRNAs) [6]. The term of lncRNA was introduced to distinguish the special class of ncRNA from well-known small regulatory RNAs (i.e. miRNAs and siRNAs). lncRNAs are generally longer than 200 nucleotides [3, 7, 8]. Recent studies have shown that lncRNAs may act as important *cis- *or *trans-*regulators in various biological processes. Mutations in lncRNAs are related with a wide range of diseases, especially cancers and neurodegenerative diseases. Even so, the functions and molecular mechanisms of most lncRNAs are unknown. Though several computational methods have been developed to predict the functions of lncRNAs, it still remains a challenging task, partly owing to the lack of conservation in both the sequence and secondary structures of lncRNAs [9–11]. In this paper, we will summarize the recent progresses and challenges in the identification, molecular mechanism, and function prediction of lncRNAs. 

## 2. Definition and Classification of lncRNA

The definition of lncRNA is based on two criteria, the size and the lack of protein-coding potential. In this paper, lncRNA refers to nonprotein-coding RNA longer than 200 nt [7, 10–12], which distinguishes it them from mRNA and small regulatory RNA in a relatively satisfying way [11, 13]. Depending on their relationships with the nearest protein-coding genes, lncRNAs can be classified in three different ways [12, 14, 15]: (1) *sense or antisense:* lncRNAs that are located on the same strand or the opposite strand of the nearest protein-coding genes [16]; (2) *divergent or convergent: *lncRNAs that are transcribed in the divergent or convergent orientation compared to that of the nearest protein-coding genes [12]; (3) *intronic or intergenic: *lncRNAs that locate inside the introns of a protein-coding gene, or in the interval regions between two protein-coding genes [12, 17]. 

## 3. Identification of lncRNA

To identify lncRNAs, the first step is to obtain all transcripts including ncRNAs and mRNAs in cells, and then to distinguish lncRNAs from mRNAs and other types of ncRNAs. Traditional technologies, such as microarray, focus on the identification of protein-coding RNA transcripts. New technologies, such as RNA-Seq, are not limited to the identification of protein-coding RNA transcripts, and have led to the discovery of many novel ncRNA transcripts. The discrimination between lncRNAs and other small regulatory ncRNAs depends on their length. However, the length information alone is not enough to separate lncRNAs from mRNAs, and other criteria are needed for this purpose. Below, we will first briefly introduce new technologies in identifying RNA transcripts, especially ncRNA transcripts. Then, we will review current methods to distinguish lncRNAs from mRNAs. 

### 3.1. Experimental Methods in Identifying lncRNA

**Microarray**

Traditional microarray technologies use predefined probes to determine the expression level of mRNA transcripts and are not appropriate to identify lncRNAs. However, it has been found that a few previously defined mRNAs or some probe sequences actually are lncRNAs; thus, former microarray datasets can be reannotated to study the expression of lncRNAs [60]. With more and more lncRNAs discovered, new probes specific for lncRNAs can be designed. For example, Babak et al. designed probes from conserved intergenic and intragenic region to identify potential ncRNA transcripts [61]. However, microarray is not sensitive enough to detect RNA transcripts with low-expression level. Thus the use of microarray to identify lncRNAs is limited due to the low expression level of many lncRNAs.

**SAGE and EST**

SAGE (serial analysis of gene expression) technology produces large numbers of short sequence tags and is capable of identifying both known and unknown transcripts. SAGE has been used and proved to be an efficient approach in studying lncRNAs. For example, Gibb et al. compiled 272 human SAGE libraries. By passing over 24 million tags they were able to generate lncRNA expression profiles in human normal and cancer tissues [62]. Lee et al. also used SAGE to identify potential lncRNA candidates in male germ cell [63]. However, SAGE is much more expensive than microarray, therefore is not widely employed in large-scale studies. EST (expressed sequence tag) is a short subsequence of cDNA, and is generated from one-shot sequencing of cDNA clone. The public database now contains over 72.6 million EST (GeneBank 2011), making it possible to discover novel transcripts. For example, Furuno et al. clustered EST to find functional and novel lncRNAs in mammalian [64]. Huang et al. used the public bovine-specific EST database to reconstruct transcript assemblies, and find transcripts in intergenic regions that are likely putative lncRNAs [65].

**RNA-Seq**

**Figure 1:**
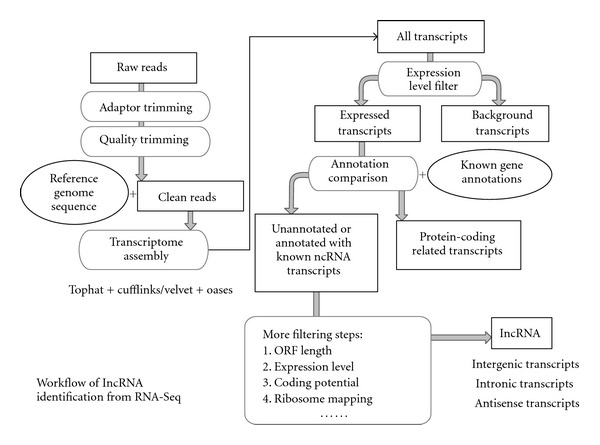
Workflow of lncRNA identification from RNA-Seq.

With the development of next generation sequencing (NGS) technologies, RNA-Seq (also named whole transcriptome shotgun sequencing) has been widely used for novel transcripts discovery and gene expression analysis. Compared to traditional microarray technology, RNA-Seq has many advantages in studying gene expression. It is more sensitive in detecting less-abundant transcripts, and identifying novel alternative splicing isoforms and novel ncRNA transcripts. The basic workflow for lncRNA identification using RNA-Seq is shown in Figure 1. RNA-Seq is currently the most widely used technology in identifying lncRNAs. For example, Li et al. applied RNA-Seq to identify lncRNAs during chicken muscle development [66]. Nam and Bartel integrated RNA-Seq, poly (A)-site, and ribosome mapping information to obtain lncRNAs in *C. elegans* [16]. Pauli et al. performed RNA-Seq experiments at eight stages during zebrafish early development, and identified 1133 noncoding multiexonic transcripts [67]. Prensner et al. used RNA-Seq to study lncRNA in human prostate cancer from 102 prostate tissues and cell lines, and concluded that lncRNAs may be used for cancer subtype classification [68].

**RNA-IP**

RNA-IP (RNA-immunoprecipitation) is a new method developed to identify lncRNA that interacts with specific protein. Antibodies of the protein are first used to isolate lncRNA-protein complexes. Then, cDNA library is constructed followed by deep sequencing of interacting lncRNAs. Using RNA-IP, Zhao et al. discovered a 1.6-kb lncRNA within Xist that interacts with PRC2 [69].

**Chromatin Signature-Based Approach**

The above-mentioned methods target on RNA transcripts directly. In contrast, chromatin signature-based approach uses chromatin signatures, such as H3K4me3 (the marker of active promoters) and H3K36me3 (the marker of transcribed region), to study actively transcribed genes including lncRNAs. In this approach, ChIP-Seq is used to generate genome-wide profiles of chromatin signatures [70], and the transcribed regions are mapped in the genome, where lncRNAs are determined and studied. For example, Guttman et al. identified 1,600 large multiexonic lncRNAs that are regulated by key transcription factors such as p53 and NFkB [71]. The advantage of this approach is its directness in investigating the mechanisms that regulate lncRNA expression.

### 3.2. Computational Methods in Identifying lncRNA

**ORF Length Strategy**

Unlike protein-coding genes, the start codons and termination codons in lncRNAs tend to distribute randomly. As a result, the ORF length of lncRNAs can hardly extend to over 100 from a probabilistic point of view. Based on this principle, one way to discriminate lncRNAs from mRNAs is by ORF length. For example, the FANTOM project used a maximum ORF length cutoff of 100 codons to differentiate noncoding RNAs from mRNAs [72]. However, some lncRNAs are known to have ORFs longer than 100 codons, while some protein coding genes have fewer than 100 amino acids, such as RCI2A gene in *Arabidopsis* which encodes a protein of 54 amino acids [73]. Thus, this approach may cause misclassification. To overcome the drawbacks of methods based on ORF length, Jia et al. utilize a comparative genomics method to refine ncRNA candidates. They defined the RNA sequences as ncRNAs only if the cDNAs have no homologous proteins longer than 30 amino acids across the mammalian genomes [7]. However, this method relies largely on the completeness of the databases. Therefore, deficiency in protein coding annotation may cause misclassification of lncRNAs as well.

**Sequence and Secondary Structure Conservation Strategy**

Compared to protein coding genes, noncoding genes are generally less conservative, meaning they are more inclined to mutate [21, 67]. Thus, measuring the coding potential is considered a way of identifying lncRNAs. Codon Substitution Frequency (CSF) is one of the criteria. For example, Guttman et al. used the maximum CSF score to assess the coding potential of a RNA sequence [71]. Clamp et al. and Lin et al. further combined CSF with reading frame conservation (RFC) to discriminate lncRNAs from mRNAs [74, 75]. Other similar methods include PhyloCSF use a phylogenetic framework to build two phylogenetic codon models that can distinguish coding from noncoding regions [76]. RNAcode combines amino acid substitution with gap patterns to assess the coding potential [77]. There are also methods that explore the conservation of RNA secondary structures to identify lncRNAs, including programs QRNA [78], RNAz [79], and EvoFOLD [80]. However, this approach is limited by lack of common conserved secondary structures specific for lncRNAs.

**Machine Learning Strategies**

Owing to the complex identities of lncRNAs, recently an increasing number of machine learning-based methods have been developed to integrate various sources of data to distinguish lncRNAs from mRNAs.  Table 1 summarizes the machine learning methods and the features used to train the model for identifying lncRNAs. For instance, CONC utilizes a series of protein features such as amino acid composition, secondary structure, and peptide length, to train a SVM model that distinguishes lncRNAs from mRNAs [18]. CPC (Coding Potential Calculator) also uses SVM for modeling and extracting sequence features and the comparative genomics features to assess the coding potential of transcripts [19, 20]. Lu et al. developed a machine learning method that integrates GC content, DNA conservation, and expression information to predict lncRNAs in *C. elegans* [21].

Although the above-described methods have shown their effectiveness in identifying lncRNAs, exceptional cases still remain. For instance, whether an RNA transcript is translated or not may be changeable during the course of evolution. As an example, *Xist*, a well-known lncRNA, evolves from a protein-coding gene [81]. Besides, some genes are bifunctional, and both the coding and noncoding isoforms exist. The steroid receptor RNA activator (SRA) was characterized as a noncoding RNA previously but the coding product was detected later [82]. Such ambiguity will be clarified when more about lncRNAs are known.

## 4. lncRNA Function

lncRNAs have once been thought as the “dark matter” of the genome, because of our limited knowledge about their functions [83]. With more studies about lncRNAs conducted, it has become clear that lncRNAs have many specific functional features, and are likely to be involved in many diverse biological processes in cells. Rather than “dark matter,” they may act as necessary functional parts in the genome. These functional features include but are not limited to (i) lncRNAs have conserved splice junctions and introns [84]; (ii) the expression patterns of lncRNAs are tissue- and cell-specific [12, 67]; (iii) the altered expression of lncRNAs can be found in neurodegeneration, cancer, and other diseases [9, 10]; (iv) lncRNAs are associated with particular chromatin signatures that are indicative of actively transcribed genes [11, 85]. Below, we will briefly summarize the cellular functions of lncRNAs and molecular mechanisms of their functions. 

### 4.1. Cellular Functions of lncRNA

With thousands of lncRNAs identified in mammals and other vertebrates [16], a few lncRNAs have been extensively studied, which have shed light on their possible functions. Firstly, lncRNAs are involved in various epigenetic regulations through recruitment of chromatin remodeling complexes to specific genomic loci, such as Xist, Air, and Kcnq1ot1 [22, 43]. Secondly, lncRNAs can regulate gene expression by interacting with protein partners in biological processes like protein synthesis, imprinting (Kcnq1ot1, Air), cell cycle control (TERRA), alternative splicing (MALAT1), and chromatin structure regulation (DNMT3b, PANDA) [9, 10, 38, 71, 85–89]. Thirdly, lncRNAs are involved in enhancer-regulating gene activation (eRNAs), in which cases they may interact directly with distal genomic regions [90]. Fourthly, some lncRNAs serve as interacting partners or precursors for short regulatory ncRNAs [91]. For example, microRNAs (miRNAs) can be generated through sequential cleavage of lncRNAs, while Piwi-interacting RNAs (piRNAs) can be produced by processing a single lncRNA transcript [88]. 

Recent studies have shown the expression of lncRNA is tissue specific. Loewer et al. studied the expression of lncRNA in global remodeling of the epigenome and during reprogramming of somatic cells to induce pluripotent stem cells (iPSCs). They found some lncRNAs have cell-type specific expression pattern [26, 92]. Loss-of-function studies on most intergenic lncRNAs expressed in mouse embryonic stem (ES) cells revealed that knockdown of intergenic lncRNAs has major consequences on gene expression patterns, which are comparable to the effects of knockdown of well-known ES cell regulators [93]. This indicated that lncRNAs might play important roles in regulating developmental process. The ENCODE project analyzed the tissue-specific expression of lncRNAs in 31 cell types, and found that many lncRNAs have brain-specific expression pattern [9, 12]. There are increasing lines of evidences that link dysregulations of lncRNAs to diverse human diseases ranging from neuron diseases to cancer [9, 10], suggesting that the involvement of lncRNAs in human diseases can be far more prevalent than previously thought [94].

### 4.2. Molecular Mechanisms of lncRNA

The precise mechanism of how lncRNAs function still remains largely unknown. Currently, there are several hypothesis about it, including (1) RNA:DNA:DNA triplex (*trans*-); (2) RNA:DNA hybrid; (3) RNA:RNA hybrid of lncRNA with a nascent transcript; (4) RNA-protein interaction (*cis-/trans-*). Although only (1), (2), and (4) have been experimentally demonstrated so far [14], it is generally thought that lncRNAs may function through the interaction with its partners, such as DNA, RNA, or protein, and serve the following roles: signal, decoy, scaffold, and guide [11, 14].  Table 2 lists lncRNAs that use different mechanisms when carrying out their functions. Below, we give examples for the above-mentioned mechanisms.

**Signal**

Some lncRNAs have been reported to respond to diverse stimuli, hinting they may act as molecular signals [12, 24, 25, 27, 35]. For example, lncRNAs can act as markers for imprinting (Air and Kcnq1ot1), X inactivation (Xist), and silencing (COOLAIR). ChIP-Seq studies showed that the gene-activating enhancers produce lncRNA transcripts (eRNAs) [29, 95], and their expression level positively correlates with that of nearby genes, indicating a possible role in regulating mRNA synthesis. This is supported by a recent Loss-of-Function study that found the knockdown of 7 out of 12 lncRNAs affects expression of their cognate neighboring genes [8].

**Decoy**

lncRNA can function as molecular decoy to negatively regulate an effector. Gas5 contains a hairpin sequence motif that resembles the DNA-binding site of the glucocorticoid receptor [31]. It can serve as a decoy to release the receptor from DNA to prevent transcription of metabolic genes [14]. Another example is the telomeric repeat-containing RNA (TERRA). It interacts with the telomerase protein through a repeat sequence complementary to the template sequence of telomerase RNA [11, 34].

**Guide**

Upon interaction with the target molecular, lncRNA may have the ability to guide it into the proper position either in *cis *(on neighboring genes) or in *trans *(on distantly located genes). The newly found eRNAs appear to exert their effects in *cis *by binding to specific enhancers and actively engaged in regulating mRNA synthesis [11, 29]. HOTAIR and HOTTIP are transcribed within the human *HOX *clusters, and serve as signals of anatomic positions by expressing in cells that have distal and posterior positional identities; they both require the interacting partners to be properly localized to the site of action [6]. In this process, chromosomal looping of the 5′ end of *HOXA *brings HOTTIP into the spatial proximity of multiple *HOXA *genes, enforcing the maintenance of H3K4me3 and gene activation [14]. This long-range gene activation mechanism suggests that chromosome looping plays a central role in delivering lncRNA to its site of action [11, 45].

**Scaffold**

Recent studies found that several lncRNAs have the capacity to bind more than two protein partners, where the lncRNAs serve as adaptors to form the functional protein complexes. The telomerase RNA TERC (TERRA) is a classic example of RNA scaffold, and is essential for telomerase function. *HOTAIR* binds the polycomb complex PRC2 to exert its “signal” function. A recent study found that the 3,700 nt of HOTAIR also interact with a second complex consisting of LSD1, CoREST, and REST to antagonize gene activation, further emphasizing its important role as the scaffold of the functional complex [11, 51].

**Cis- and Trans-Action of lncRNAs**

lncRNAs can be classified as *cis-* or *trans-*regulators depending on whether it exerts its function on a neighboring gene on the same allele from which it is transcribed [96]. It was considered that many lncRNAs act as *cis*-regulators, as the expression of lncRNA is significantly correlated with their neighboring protein-coding genes [97, 98]. However, recent studies have questioned that the positive correlation between lncRNAs and their neighboring genes may be due to shared upstream regulation (such as, lincRNA-*p21* [24] and lincRNA-*Sox2* [6]), positional correlation (such as, HOTAIR [6]), transcriptional “ripple effects” [98], and indirect regulation of neighboring genes, instead of the effects of *cis*-regulation. This was supported by the fact that knock down of different number of lncRNAs had little effect on the expression of neighboring genes [96]. In general, it has been accepted that some lncRNAs are *cis*-regulators [99, 100], while the vast majority may function as *trans*-regulators [6, 11, 93]. Recently, some *cis*-regulating lncRNAs were found to have the capacity to act in *trans* [33, 101, 102], highlighting the complexity of lncRNAs.

Although substantial research progresses have been made since the discovery of lncRNAs, it still remains a challenge to understand the functions of lncRNAs. One reason is, unlike protein-coding genes whose mutations may result in severely obvious phenotypes, mutations in lncRNAs often do not cause significant phenotypes [85]. It is likely that lncRNAs may function at specific stage of development process or under specific conditions, and thus condition-specific studies of lncRNAs' phenotypes may be necessary. With more omics data about lncRNAs accumulating, computational prediction of the function of lncRNAs can help to design experiments to accelerate the understanding of lncRNAs.

## 5. lncRNA Database

The current lncRNA databases are summarized in Table 3. lncRNAdb is an integrated database specific for lncRNAs, including annotation, sequence, structural, species, and function categories of lncRNAs [55]. NONCODE is a database about ncRNAs that have been experimentally confirmed. It covers almost all published 73,272 lncRNAs in human and mouse; it also includes expression profiles of lncRNAs and their potential functions predicted from Coding-Noncoding coexpression network (see below) [56]. LNCipedia is another integrated lncRNA database, which includes 21,488 annotated human lncRNAs. It contains lncRNAs information about the coding potential, secondary structure, and microRNA binding sites [57]. fRNAdb and NRED are databases for ncRNAs including lncRNAs [58, 59]. The above databases provide great convenience for further analysis and applications of lncRNAs. 

## 6. Function Prediction of lncRNA

Computational prediction of lncRNA functions is still at its early development stage. Unlike protein-coding genes whose sequence motifs are indicative of their function, lncRNA sequences are usually not conserved and do not contain conserved sequence motifs [103, 104]. The secondary structures of lncRNA are also not conserved [105]. Thus, it is difficult to infer the function of lncRNAs based on their sequences or secondary structures alone. Since current knowledge suggests that lncRNAs function by regulating or interacting with its partner molecular, current methods focus on exploring the relationships between lncRNAs and protein-coding genes or miRNAs. Below, we will describe several current approaches for predicting the functions of lncRNAs. 

### 6.1. Comparative Genomics Approach

Although most lncRNAs are not conserved, there are lncRNAs that are conserved across species, indicating their essential functions. Amit et al. identified 78 lncRNAs transcripts conserved in both human and mouse, and found 70 are either located within or close (<1000 nt distance) to a coding gene that is also conserved in the two genomes [106]. They assumed these lncRNAs might have close functional relationships with the nearby coding genes. However, this approach is limited because of the poor conservation of lncRNAs and cannot be applied at genome scale. 

### 6.2. Coexpression with Coding Genes Approach

Many studied lncRNAs play important regulatory roles, and it is likely that lncRNAs regulating a specific biological process may be coexpressed with the genes involved in the same process. Thus, identifying coding genes that are coexpressed with lncRNAs may help to infer the function of lncRNAs. Based on this assumption, Guttman et al. developed a coexpression based method to predict lncRNAs functions at genome scale [71]. For each lncRNA, they ranked coding genes based on their coexpression level with the lncRNAs, and then performed a Gene Set Enrichment Analysis (GSEA) for the top-ranked genes to identify enriched functional terms corresponding to the lncRNAs. Out of 150 lncRNAs subjected for experimental validation, 85 exhibited the predicted functions, proving the effectiveness of using the coexpressed coding genes to infer the function of lncRNAs from their coexpressed coding genes. According to their predictions, lncRNAs participate in a rather wide range of biological processes such as cell proliferation, development, and immune surveillance. Andrea et al. employed a similar approach to predict the function of lncRNAs during zebrafish embryogenesis [67].

Liao et al. furthered the coexpression idea by constructing a coding-noncoding (CNC) gene coexpression network [107]. In contrast to the GSEA method that collects coding genes coexpressed for each lncRNA, the CNC method considers not only the coexpression between lncRNAs and coding genes, but also within lncRNAs group and coding gene group. When predicting the function of lncRNAs, the CNC method employs two different approaches: the hub-based and the network-module-based. In the hub-based approach, functions are assigned to each lncRNA according to the functional enrichment of its neighboring genes. In the network-module-based approach, Markov cluster algorithm (MCL) is used to identify coexpressed functional module in the CNC network; then functions of the module are transferred to the lncRNAs inside the module. Liao et al. applied the CNC method to annotate the functions of 340 mouse lncRNAs, and found these lncRNAs function mainly in organ or tissue development, cellular transport, and metabolic processes. 

### 6.3. Interaction with miRNAs and Proteins Approach

Recent analysis found that lncRNAs share a synergism with miRNA in the regulatory network [108, 109]. It is likely that some lncRNAs function by binding miRNA. Therefore, identifying well-established miRNAs that bind lncRNAs may help to infer the function of lncRNAs. Jeggari et al. developed an algorithm named miRcode that predicts putative microRNA binding sites in lncRNAs using criteria such as seed complementarity and evolutionary conservation [110]. Jalali et al. constructed a genome-wide network of validated RNA mediated interactions, and uncovered previously unknown mediatory roles of lncRNA between miRNA and mRNA (Saakshi Jalali, arXiv preprint). Besides the interaction with miRNA, the interaction of lncRNAs with proteins can also be explored to predict their functions. Bellucci et al. developed a method called “catRAPID” that correlates lncRNAs with proteins by evaluating their interaction potential using physicochemical characteristics, including secondary structure, hydrogen bonding, van der Waals, and so forth [111]. However, unlike the coexpression based approach, the above two approaches were successful in only a number of lncRNAs, partly because the mechanism of how lncRNAs interact with miRNAs and proteins still remains unclear. 

### 6.4. Challenges

Computational prediction of lncRNA functions is still at its primary stage. As the sequence and secondary structure of lncRNAs are generally not conserved, function prediction of lncRNAs mainly relies on their relationships with other moleculars, such as protein coding genes, miRNAs, and proteins. However, the molecular mechanism of how lncRNA function by interacting with other molecular remains largely unknown, making it difficult to develop computational methods to precisely predict the functions of lncRNAs. On the other hand, there are currently only a small number of lncRNAs whose functions are well understood, which makes it difficult to validate and optimize computational algorithms for predicting lncRNA functions. Finally, unlike protein-coding genes that have systematic functional annotation systems, there lacks an annotation system for lncRNA functions, making it difficult to evaluate computational algorithms for function prediction. Nevertheless, the success of predicting lncRNAs using the coexpression based approach has shown promises. With more functional genomics data about lncRNAs available in the near future, more powerful and accurate methods will be developed to help decipher the functions of lncRNAs. 

## 7. Perspectives

It has been widely accepted that lncRNAs play important functional roles in cell, though the molecular mechanism of how lncRNAs function remains to be unraveled. In this paper, we have described several currently proposed models about the molecular mechanism of lncRNA functions. One commonality about these models is that lncRNAs function through the interaction with other molecular, including DNA, RNA, and proteins. Given the abundance of lncRNAs in genome, it is likely that the interaction between lncRNAs and other moleculars may be specific. This thus raises the possibility of developing novel methods to target certain lncRNA for gene-specific regulation. However, phenotypic studies of lncRNAs suggested that knockdown of many lncRNAs does not result in obvious phenotypes, making it difficult to understand their functions. Computational prediction of lncRNAs can provide hypothesis about the functions of lncRNAs, and help to design experiments to test them under specific conditions. Yet, it remains a significant challenge to develop effective methods to accurately infer the lncRNA functions, owing to the lack of detailed information about the molecular mechanisms of lncRNAs. In order to develop powerful computational methods, more studies about the derivation of lncRNAs, the molecular mechanism of lncRNAs and tissue-specific, or development-specific expression about lncRNAs are necessary.

## Figures and Tables

**Table 1:** Machine-learning methods for identifying lncRNAs.

Method	Features	Algorithm	References
	Peptide length		
	Amino acid composition		
	Hydrophobicity		
CONC	Secondary structure content	SVM	[18]
	Percentage of residues exposed to solvent		
	Sequence compositional entropy		
	Number of homologs obtained by PSI-BLAST		
	Alignment entropy		

	ORF prediction quality		
CPC	Number of homologs obtained by BLASTX	SVM	[19, 20]
	Alignment quality		
	Segment distribution		

Lu et al.	RNA-seq experiments Tilling arrays poly-A + RNA-seq experiments poly-A + tilling arrays GC content DNA conservation Predicted protein sequence conservation Predicted secondary structure free energy Predicted secondary structure conservation	Naïve Bayes Bayes Net Decision Tree Random Forest Logistic Regression SVM	[21]

**Table 2:** Function classification of lncRNAs.

Archetype	lncRNA name	Length	Target	Function	*cis*-/*trans*-	References
Signal	KCNQ1ot1, Air, Xist	91 kb, 108 kb, ~17 kb	G9a, PRC, YY1	Transcriptional silencing of multiple genes; X inactivation (XCI)	*cis- *	[11, 14, 22, 23]
	HOTAIR, Frigidair, HOTTIP,	2.2 kb, N.A., 3.7 kb	LSD1-CoREST	Signals of anatomic position,	*trans*-	[6, 11, 14]
	lincRNA-p21, PANDA	3 kb; 1.5 kb	hnRNP-K	p53 targets in response to DNA damage	*trans*-	[14, 24, 25]
	lincRNA-RoR	2.6 kb	Oct4, Sox2, Nanog	Pluripotency-associated	N.A.^b^	[11, 26]
	COOLAIR, COLDAIR	Multiple spliced: 400 bp/750 bp; ~1.1 kb	FLC, PRC2	Combinatorial transcriptional regulation	N.A.	[27, 28]
	eRNA	Various sizes	MLL-WDR5, TFs^a^	Promotes mRNA synthesis	*cis*-	[29, 30]
	Gas5	~7 kb	Glucocorticoid receptor	Represses the glucocorticoid receptor	N.A.	[31]
	1/2-sbsRNAs	N.A.^c^	SMD	Formation of STAU1 binding sites	N.A.	[32]

Decoys	DHFR-Minor	7.3, 5.0, 1.4, and 0.8 kb	TFIIB	Inhibits assembly of the preinitiation complex	N.A.	[33]
	TERRA	Various sizes	Telomerase	Regulation and protection of chromosome ends	N.A.	[34]
	PANDA	1.5 kb	NF-YA	Inhibits expression of apoptotic genes	*trans- *	[35]
	*PTENP1 *	~3.9 kb	PTEN	Sequestration of miRNAs	N.A.	[36, 37]
	MALAT1	~7 kb	SR splicing factors	Alters pattern of alternative splicing	N.A.	[38, 39]

Guides	Xist	~17 kb	PRC2, YY1	Inactives X chromosome	*cis*-	[14, 40–42]
	Air, COLDAIR	108 kb,	G9a, PRC2	Silences transcription, affects histone acetylation and methylation states	*cis*-	[28, 43, 44]
	HOTTIP	~3.8 kb	MLL-WDR5	Chromosomal looping, chromatin modifications	*cis*- (looping)	[11, 45]
	HOTAIR	2.2 kb	LSD1-CoREST	Alters and regulates epigenetic states	*trans- *	[14, 46, 47]
	Jpx	Multiple isoforms	polycomb complex^a^	Activation of Xist RNA on the inactive X	*trans- *	[11, 48]
	lincRNA-p21	3 kb	hnRNP-K^a^	p53 targets in response to DNA damage	*trans- *	[11, 24]

Scaffold	TERC	Various sizes	TERT	Telomerase catalytic activity	*trans- *	[49, 50]
	HOTAIR	2.2 kb	PRC2, LSD1, CoREST, REST	Demethylates histone H3 on K4 to antagonize gene activation	*trans- *	[46, 51]
	ANRIL	Multiple spliced: 3.9 kb/34.8 kb	PRC1, PRC2	Contributes to the functions of both PRC1 and PRC2 proteins	*trans- *	[52, 53]
	Alpha Satellite Repeat LncRNA	N.A.	SUMO-HP1	Molecular scaffold for the targeting and local accumulation of HP1	N.A.	[11, 54]

^a^Not yet understood.

^b^Not clearly referred as *cis-*action.

^c^No length data available in all six databases listed in Table 3.

**Table 3:** List of lncRNA databases.

Tools	Source	Description	Reference
lncRNAdb	http://www.lncrnadb.org/	Contain comprehensive list of lncRNAs in eukaryotes, and mRNAs with regulatory roles	[55]
NONCODE	http://noncode.org/	Integrative annotation of noncoding RNA (73,372 lncRNAs)	[56]
LNCipedia	http://www.lncipedia.org/	21 488 annotated human lncRNA transcripts with secondary structure information, protein coding potential, and microRNA binding sites	[57]
fRNAdb	http://www.ncrna.org/frnadb/	A large collection of noncoding transcripts including annotated/unannotated sequences from H-inv database, NONCODE, and RNAdb	[58]
NRED	http://jsm-research.imb.uq.edu.au/nred/cgi-bin/ncrnadb.pl/	Noncoding RNA Expression Database	[59]
